# Topographic Mesocortex-Amygdalar Projections Expand the Molecular and Functional Model of the Limbic System

**DOI:** 10.1007/s12035-026-06153-8

**Published:** 2026-08-25

**Authors:** Luis Puelles, Gloria Fernández, Elena Garcia-Calero

**Affiliations:** 1 Departamento de Anatomía Humana y Psicobiología, Facultad de Medicina, Universidad de Murcia, Campus de Ciencias de la Salud, 30120 Murcia, Spain; 2 Instituto Murciano de Investigación Biosanitaria IMIB-Pascual Parrilla, 30120 Murcia, Spain

**Keywords:** Limbic connectome, Limbic mesocortex, Amygdalar radial units, Emotional evaluation, Mental disorders, *Self*

## Abstract

The limbic connectome is responsible for emotional evaluation and behavioral response to external and internal stimuli. Anxiety, depression, phobias and posttraumatic stress are mental disorders caused by the dysfunction of this system. We compared the pattern of anterograde projections of the full limbic mesocortical ring and the isocortex onto the glutamatergic/pallial amygdala. We used the amygdalar radial model, confirmed by transcriptomic studies, as well as anterograde connectivity experiments published in the Allen Brain Mouse Connectivity Atlas public database (https://connectivity.brain-map.org/). The radial amygdalar model subdivides the glutamatergic amygdala into radial histogenetic-molecularly defined superdomains, domains and subdomains, each stratified in periventricular, intermediate and superficial nuclei. The mesocortical ring projections center onto the *basolateral* radial amygdalar subdomain and constitute a fundamental pathway in the limbic connectome, whereas the isocortical amygdalar input centers on the *lateral* radial unit. The mesocortical projections show a medial to lateral topographical distribution between the *medial* and *lateral* subdivisions of the *basolateral* amygdala. Intra-amygdalar connections suggest that computations in the *lateral* and *anterior* amygdalar radial domains add layers of complexity to the functions of the *basolateral* unit. The medial posterior orbitary cortex, proposed previously as the integrative node of the mesocortical ring, projects onto the *medial basolateral* amygdalar subunit, pointing to this neural structure as an important hub of the limbic system.

**Supplementary Information:**

The online version contains supplementary material available at 10.1007/s12035-026-06153-8.

## Introduction

Cortico-amygdalar projections represent collectively an important computational link within the limbic connectome. The mesocortical ring (defined molecularly in Puelles et al. [1]) is reportedly involved in the evaluation of perceptions, motivations, and intentions. The associative isocortex instead elaborates information about perceptions and behavioral planning. Both inputs modulate amygdalar functional outputs reaching subcortical limbic response centers and feeding back on some parts of the cortex. Recent redefinition of mouse limbic cortex as a complete ring (cited above) requires closer study of these connections in order to understand better the functioning of the cortico-amygdalar circuit within the limbic connectome. Such analysis may throw light upon possible dysfunctions underlying mental diseases such as anxiety, post-traumatic stress, chronic phobias, or depression.

The quest of deeper understanding of the limbic system led our research group to elaborate new *molecularly informed* models of both the limbic cortex [1–3] and the pallial amygdala [4–6]. Thinking within the modern theory of cortical concentric rings (the notion that concentric allocortex and mesocortex rings surround a central isocortical island; reviewed in Puelles et al. [1, 2]), we visualized the limbic cortex as the inner mesocortical ring around the isocortex, whose diverse areal sectors (e.g., cingulate, posterior orbital, insular, etc.) are co-defined by a shared differential molecular profile [1, 3]. Allocortical areas forming an outer cortical ring (hippocampal and olfactory cortices) are also sharply different molecularly (see Yao et al. [7]) and are often excluded from the ‘limbic’ functional and structural category (i.e., there was a confusing mixture of allocortical and mesocortical areas in the historical versions of this concept). The mesocortical ring is currently divided into the following sectors: medial and ventrolateral posterior orbitary (POrbM, POrbVL), prelimbic (PL), cingulate (Cing), parasplenial (PaSp), postsplenial (PoSp), postrhinal (PoRh), perirhinal (PeRh) and insular (Ins) cortices [1]. We have recently found that these mesocortical sectors are richly interconnected mutually (within the mesocortical limbic ring), and the POrbM cortex stands out as the hierarchically dominating structure, being bidirectionally connected with all the other sectors [8].

On the other hand, conventional neuroanatomic schemata of the mammalian amygdala are handicapped by its peculiar, deformed morphogenesis at the tip of the temporal lobe, causing that the histogenetic radial (ventriculo-pial) dimension is conventionally disregarded. We conceived a topologically invariant radial model of the mouse glutamatergic (pallial) amygdala that can be extrapolated to any mammal. This model contemplates how the neuroepithelial amygdalar primordium develops histogenetically (i.e., via proliferation, neurogenesis, and differentiation), to produce specific stratified nuclei in an outside-in neurogenetic pattern displaying a handful of molecularly distinct adjacent radial histogenetic domains. This model conceives the glutamatergic amygdala as subdivided into two radial superdomains (hypothalamus-like versus cortex-like territories), differentiated respectively by expression of the glutamatergic markers *Slc17a6* and *Slc17a7* (Hy-A; Cx-A [5, 6]). The Hy-A divides into the *anterior* (ant) and *medioventral* (mev) radial domains. The Cx-A contains the *lateral* (lat), *basal* (bas), and *posterior* (post) radial units [4–6]. Consistently with transcriptomic analysis, the large *basal* domain is subdivided into *basolateral* (bl) and *basomedial* (bm) subdomains; the *basolateral* subunit can be further divided into *lateral basolateral* (bll) and *medial basolateral* (blm) radial microdomains [4–6]. Classic and modern studies on amygdalar structure and connections (e.g. [9]) largely without taking into account the relative positions of the ventricular and pial surfaces of the amygdalar complex. The conventional sectioning planes through the amygdala used in atlases and other sources are usually oblique to the natural radial embryologic dimension of this nuclear complex, causing various interpretive problems. The superficial amygdalar nuclei were misconceived as corticoid or cortical components, while modern histogenetic and neurogenetic analysis suggests that the whole pallial amygdala is nuclear.

In the present study, we focused on the projections of the meso- and isocortex onto the glutamatergic amygdala, using the cited updated cortical and amygdalar *molecular models* as a basis. We found that the limbic mesocortical ring projects in an orderly pattern as a whole onto the whole *basolateral* radial amygdalar subdomain, whereas isocortex projects mainly to the *lateral* radial amygdalar domain. Given the novel significance of the integrative processing performed by the full mesocortical ring via its intrinsic multiple connections [8], we suggest that the main cortical input to the pallial amygdala is the limbic one (the *lateral* amygdalar unit is known to project itself also to the *basolateral* subunit, suggesting that isocortical projections have a lower weight in this circuit [10, 11]). Amygdalar mesocortical inputs as a whole show a medial-to-lateral distribution pattern onto the *medial* and *lateral basolateral* radial subdivisions. In some cases, sagittal-plane analysis of the Allen Brain Institute experimental data indicates that single mesocortical injections distribute terminals selectively throughout the postulated radial domains of the amygdala, along the ventriculo-pial dimension. Such observations corroborate tenets of the radial pallial amygdalar model. The *medial basolateral* radial subunit (blm) receives the input from the hierarchically dominating POrbM mesocortex sector and may thus have particular relevance as an important (previously unrecognized) amygdalar hub in this system. These Results suggest new functional aspects in a modern model for the limbic system.

## Material and Methods

### Allen Brain Connectivity Atlas

To develop this study, we used the Allen Brain Mouse Connectivity Atlas (ABMCA) (https://brain-map.org/our-research/connectivity).

To explore the connections of the mesocortical ring on the pallial amygdala, we used the ring-shaped selected collection of injection points in the mesocortex previously proposed and validated in Puelles and Garcia-Calero [8]. The chosen injection sites were interpreted by us as corresponding to mesocortical loci by comparison with our previous molecular definition of this ring-shaped cortex band around isocortex, using several dozen differential gene markers [1, 3] (initially some 40 markers were found that later expanded into 83 [3]). The injection sites were individually classified in the ABMCA as follows: VISC (visceral area), VISpl (posterolateral visual area), VISpm (posteromedial visual area), VISpor (postrhinal area), ACA (anterior cingulate area), PL (prelimbic area), ILA (infralimbic area), ORB (orbital area), AI (agranular insular area), RSPagl (retrosplenial area, lateral agranular part), RSPv (retrosplenial area, ventral part), PERI (perirhinal area), ECT (ectorhinal area). A number of the Allen injections marked as VISpl, VISpm, VISpor were classified by us as actually lying within the mesocortical ring sectors adjacent to these isocortical areas (PoRh, PoSp, PaSp; note these areas do not exist yet in the Allen cortex ontology) and were thus selected for our study; obviously, many other truly isocortical VIS injections were correctly classified and these were of no interest for our study. We analyzed the projections of these mesocortical regions onto pallial amygdala using the structural framework of the amygdalar radial model [4]. The Results are summarized in Table 1. The cases are identified by a *cortical areal site abbreviation tag* referring to the mesocortical sectors defined in Puelles et al. [1], followed by a *hyphen* and the corresponding *injection volume* indicated in the ABMCA in mm^3^. We reclassified positionally those cases that seemed incorrectly mapped in the Allen Brain database (compared to our molecularly defined mesocortex model [8]). Our mesocortical nomenclature of limbic cortical sectors refers to POrbM (medial posterior orbital), PL (prelimbic), Cing (cingulate), PaSp (parasplenial), PoSp (postsplenial), PoRh (postrhinal), PeRh (perirhinal), EcRh (ectorhinal), Ins (insular), and POrbVL (ventrolateral posterior orbital).

**Table 1:** Connections of the mesocortical ring and isocortex on the pallial amygdala

**POrbM**	**Target**	**PL**	**Target**	**Cing**	**Target**
ORBm-0.350	blm	PL-0.391	blm	ACAv-0.334	blm
ORBm-0.288	blm	PL-0.477	blm	ACAd-0.247	low blm
ORBm-0.283	blm	PL-0.424	blm	ACAd-0.432	low blm
ORBm-0.200	blm	PL-0.260	blm	ACAd-0.227	low blm
ORBvl-0.380	blm	PL-0.242	blm	ACAv-0.222	low blm
ORBm-0.211	blm	PL-0.258	blm, mev, post	ACAv-0.229	low blm
		ILA-0.484	blm, mev	PL-0.211	low blm
		ILA-0.205	blm, mev, post	ACAv-0.392 ACAd-0.239 ACAd-0.205 ACAd-0.274	No amygdalar labelling
		ORBm-0.208	blm		
**PaSp**	**Target**	**PoSp**	**Target**	**PoRh**	**Target**
RSPagl-0.308	low blm	RSPagl-0.105	No amygdalar labelling	VISpor-0.400	blm, lat
RSPagl-0.275	low blm			VISpl-0.319	blm, low lat
RSPagl-0.223 VISpm-0.276 VISpm-0.236 VISpm-0.436	No amygdalar labelling				
**PeRh/EcRh**	**Target**	**Ins**	**Target**	**POrbVL**	**Target**
VISC-0.450 PeRh	bll, lat, ant, low blm	AId-0.212 agranular	bll, low blm	ORBl-0.317	bll
VISC-0.448 PeRh	bll, lat, ant, low blm, low mev, post	AIv-0.416 agranular	bll, blm, ant	ORBl-0.232	bll
AIp-0.280 PeRh	Low bll, ant, low mev, low post	AId-0.398 dis/granular	bll, low blm, ant, low mev	ORBl-0.423	low bll
VISpor-0.355 (caudal PeRh)	blm, lat	AId-0.473 dis/granular	bll, low blm	ORBvl-0.296 (+ POrbM)	blm
VISpor-0.367 EcRh	blm, lat	AId-0.243 dis/granular	bll, low blm	ORBl-0.219 ORBl-0.263 ORBvl-0.202 ORBvl-0.423 ORBvl-0.386	No amygdalar labelling
VISpor-0.360 EcRh	blm, lat				
VISpor-0.310 EcRh	blm, lat				
VISpor-0.212 EcRh	blm, lat				
VISpor-0.252 EcRh	blm, lat				
**Prefrontal/motor associative cortex**	**Target**	**Auditory** **ICx**	**Target**	**Visual ICx**	**Target**
MOs-0.237	bll	AUDp-0.423	lat, low blm	VISl-0.252	lat, blm
FRP-0.094	rostral bll	AUDp-0.334	lat, low blm	VISl-0.404	lat, blm
FRP-0.156	rostral bll	AUDp-0.397	lat, blm	VISp-0.202	lat, blm
MOs-0.483	bll	AUDp-0.259	lat, blm		
MOs-0.220	bll				
MOs-0.425	bll				
MOs-0.325	bll				
MOs-0.306	bll				
MOs-0.391	bll				

Cases chosen from Allen Brain Mouse Connectivity Atlas (https://brain-map.org/our-research/connectivity). We used the injection points in the mesocortex described in Puelles and Garcia-Calero [8], their Table 1. The locus tag for each mesocortical sector defined in Puelles et al. [1] is followed by a *hyphen* and the injection site volume in mm^3^ units. For isocortical injections, the locus tag for each isocortex is followed by a *hyphen* and the injection site volume in mm3 units. For amygdalar target, we indicated the radial domain/subdomain. Experiments with injection volumes outside the range selected were excluded from our study (as well as from the previous study of Puelles and Garcia-Calero [8])

For isocortical projection analysis, we chose source isocortical injection sites listed in the ABMCA as: FRP (frontal pole, cerebral cortex), MO (somatomotor areas), SS (somatosensory areas), AUD (auditory areas), VIS (visual areas), PTLp (posterior parietal association areas), TEa (temporal association areas). For target structures in the glutamatergic amygdala, the injection sites were listed in ABMCA as: COA (cortical amygdalar area), PAA (piriform-amygdalar area), HATA (hippocampo-amygdalar area), LA (lateral amygdalar nucleus), BLA (basolateral amygdalar nucleus), BMA (basomedial amygdalar nucleus), PA (posterior amygdalar nucleus), TR (postpiriform transition area). These results are also identified in Table 1. All the ABMCA cases in Table 1 (see also Table S1) are classified with a *cortical areal positional code tag* followed by a *hyphen* and the corresponding *injection volume in mm*^*3*^.

The Allen brain connectivity cases with their amygdalar targets were annotated in Table 1 and Table S1. In general, the experiments listed in Table 1 correspond to cases with an injection volume between 0.200 and 0.500 mm^3^ (consistent with the cases studied by Puelles and Garcia-Calero [8]).

### Animals and RNAscope in situ Hybridization

Adult mice (C57BL/6 strain, source University of Murcia) were sacrificed at the Animal Housekeeping Facility, CEIB, University of Murcia (*n* = 2, one female, one male, P56). We followed the Spanish and European regulations. Our experimental protocols were approved by the University of Murcia Committee for Animal Experimental Ethics and CARM (Autonomous Community of the Region of Murcia; Approval Code, No. A13230704/Approval Date: 20 July 2023). Before sacrifice, the animals were kept in individual cages at the CEIB, under veterinary control. Animals were anesthetized with isoflurane at 4–5% and subsequently decapitated. All experiments were performed in accordance with relevant guidelines and regulations.

We carried out in situ hybridization experiments using the following RNAscope probes: Mm-*Ntng2* (585,811-C1), Mm-*Rspo2* (402,001-C3), Mm-*Rorb* (444,271-C4), Mm-*Sema3e* (449,631-C2), Mm-*Htr2c*-C1(401,001), Mm-*Etv1* (433,281-C2), Mm-*Otof* (485,671-C3) and Mm-*Meis2* (436,371-C4). We used the RNAscope Fluorescent Multiplex Kit (RNAscope® Intro Pack for Multiplex Fluorescent Reagent Kit v2- Mm with TSA Vivid Dyes-RNAscope®, Advanced Cell Diagnostics, Cat. 323,280) following the manufacturer’s instructions for fresh tissue [5].

### Image Processing

A Leica Thunder-imager Widefield microscope was used for acquisition of digital microphotographs, employing a 20 × objective. The images were processed further using a proprietary background subtraction application (Leica ICC) to optimize the results. The digital images were processed with ImageJ (NIH, https://imagej.net/ij/), Adobe Photoshop Elements and Adobe Illustrator (version CC) software (Adobe Systems MountainView, CA, USA).

## Results

### Gene Markers for the Lateral, Basal and Anterior Amygdalar Radial Domains

**Fig. 1:**
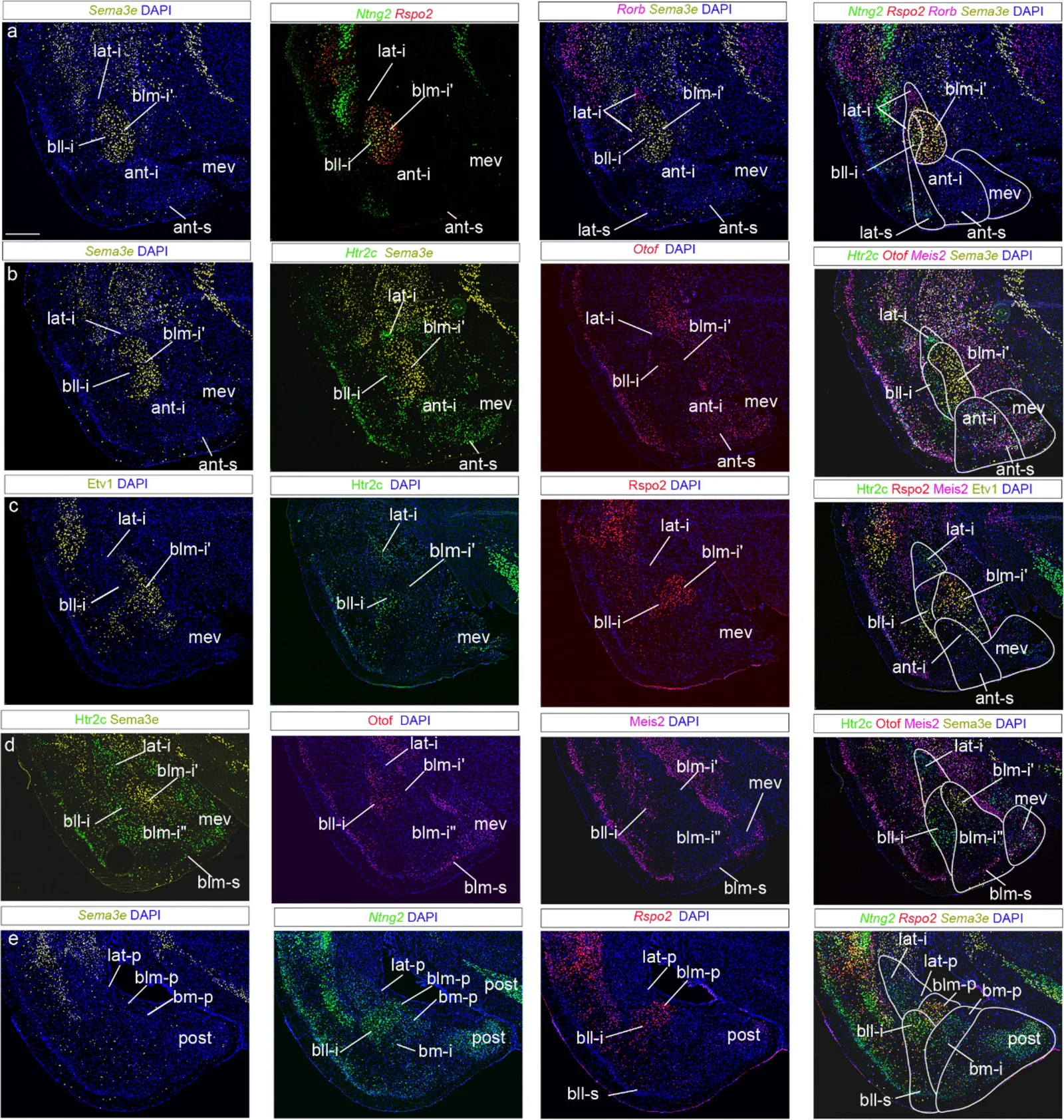
Gene markers of the *lateral*, *lateral basolateral*, *medial basolateral* and *anterior* radial domains/subdomains in pallial amygdala. Multiplexed fluorescent in situ hybridization assay for *Sema3e*, *Ntng2*, *Rspo2*, *Rorb*, *Htr2c*, *Etv1*, *Otof* and *Meis2* in the amygdalar *lateral*, *anterior*, *medioventral* and *basal* radial domains. Coronal plane from rostral (a) to caudal (e). Note that each group of 4 panels represents the same section level through the amygdala. The first three indicate the radial domains minimally with their abbreviated names. In contrast, the fourth panel shows in addition white lines delineating the full shape of the diverse radial domains visible at each section level, jointly with all superposed gene patterns. Scale bar 500 µm

In the present study, we used coronally sectioned mouse material from the Allen Brain Mouse Connectivity Atlas (ABMCA) (https://brain-map.org/our-research/connectivity), which, usefully, can be visualized also virtually in sagittal and horizontal section planes. To facilitate the understanding of the projection results, we illustrate the expression pattern of marker genes for the amygdalar radial domains published recently in our transcriptomic studies [5, 6] (Fig. 1). We focused mainly on the *lateral* (lat), *lateral* and *medial basolateral* (bll, blm), and *anterior* (ant) radial amygdalar domains/subdomains, which were the main amygdalar areas that receive projections from the iso- and mesocortex (the *posterior* radial domain receives scarce iso-meso-cortical inputs; see below). We used the same amygdalar domain abbreviations employed before in Fernández et al. [5, 6]. That is, reference to a locus within a radial amygdalar domain presents the *abbreviated name of the radial domain* connected by a *hyphen* with a letter symbolizing the radial stratum in which the marking is located, alternatively *superficial* (s), *intermediate* (i) or *periventricular* (p). Note that the ant radial domain has no periventricular stratum. In addition, the intermediate stratum of the *medial basolateral* subdomain was subdivided into *inner* -blm-i'- and *outer* -blm-i"- areas in the Figures in the paper.

At rostral levels, *Sema3e* and *Rspo2* label the *medial basolateral* subdomain, particularly its intermediate stratum (blm-i'; Fig. 1a). In addition, *Ntng2* labels the intermediate stratum of the *medial* and *lateral basolateral* subdivisions (blm-i'; bll-i; Fig. 1a). In these sections, we also observed the intermediate stratum of the *lateral* domain expressing the gene *Rorb* (lat-i; Fig. 1a). The intermediate and superficial strata of the *anterior* radial domain were negative for these genes (ant-i; ant-s; Fig. 1a). At more caudal intermediate levels, the differences between the *lateral* and *medial* subdivisions of the *basolateral* domain were shown, for example, by the expression of *Htr2c*, *Meis2* and *Otof* in bll-i, and *Etv1* in blm-i' (Fig. 1b–d). The *anterior* and *medioventral* radial domains were labelled by *Htr2c*, *Otof* and *Meis2* (ant-i; ant-s; mev; Fig. 1b–d). In these slices, we also detected distinctive molecular properties of the superficial stratum of blm (blm-s; Fig. 1d). At caudal coronal sections, we observed the periventricular strata of the *lateral* and blm units (lat-p; blm-p; Fig. 1e). The superficial stratum of bll was also distinguished in this material (bll-s, Fig. 1e). Finally, the *basomedial* subdomain, and the *posterior* radial domain were also observed at this level (bm-i; bm-p; post; Fig. 1e).

### Projections of the Mesocortical Ring onto the Pallial Amygdala

For the analysis of the mesocortical projection pattern, we used the Allen Brain Mouse Connectivity Atlas (ABMCA) (https://connectivity.brain-map.org/). The cortical injection sites and the nomenclature used in the present work to identify the injection points reproduce those published in Puelles and Garcia-Calero [8] and are explained in the Materials and Methods section of this work.

**Fig. 2:**
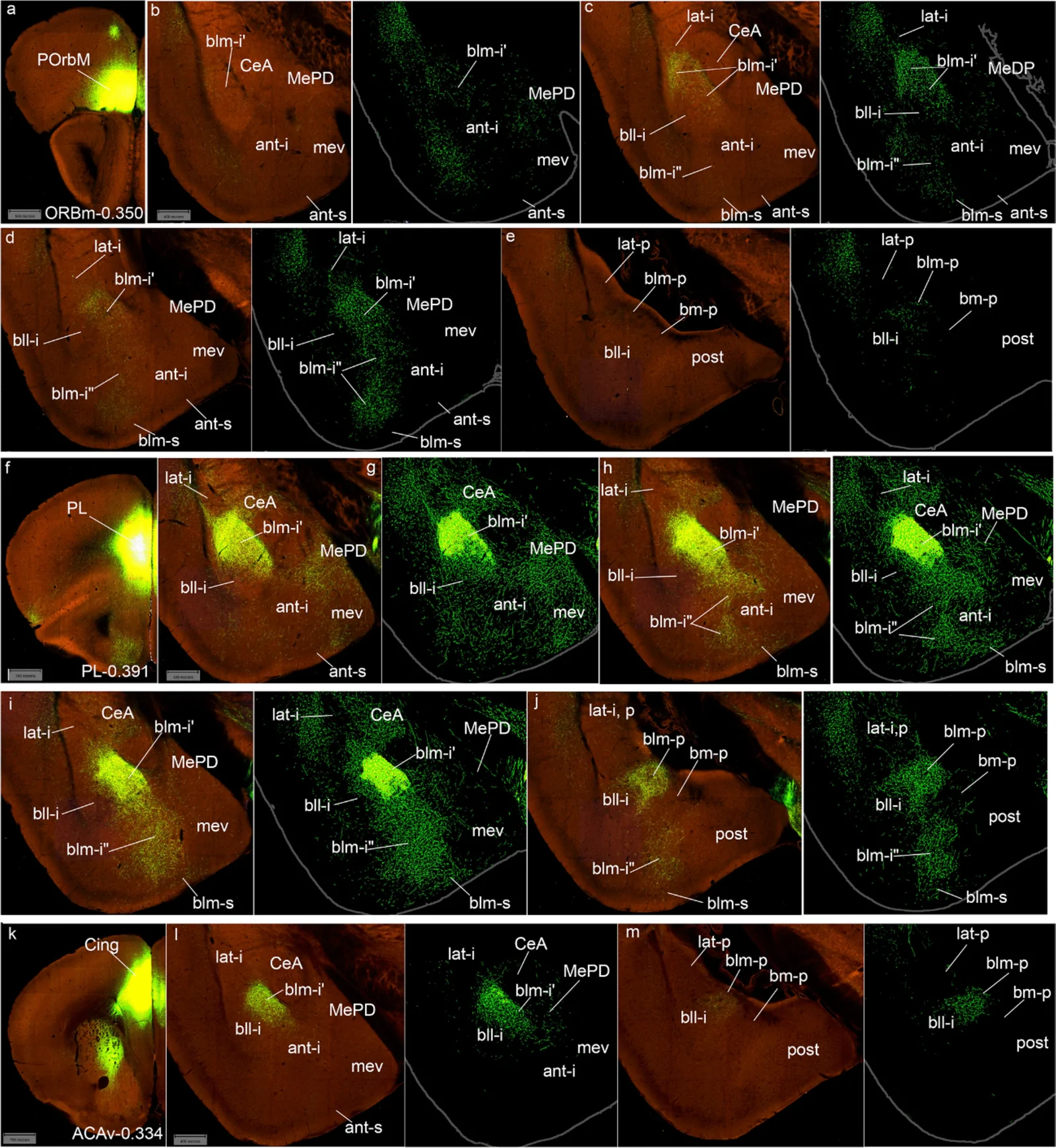
PorbM, PL and Cing cortices projections to pallial amygdala. **a** POrbM injection site, experiment ORBm-0.350. **b**–**e** POrbM projections over pallial amygdala in coronal sections from rostral to caudal. **f** PL injection site, experiment PL-0.391. **g**–**j** PL projections over pallial amygdala in coronal sections from rostral to caudal. **k** Cing injection site, experiment ACAv-0.334. **l**, **m** Cing projections over pallial amygdala in coronal sections from rostral to caudal. The material was obtained from the Allen Brain database (https://brain-map.org/our-research/connectivity), we used two-photon tomography and projection segmentation images. The scale bars were indicated in images

We analyzed section-by-section *n* = 6 cases injected in POrbM [1, 8] which labelled the amygdala (Fig. 2a–e; Table 1; Table S1). We used the ORBm-0.350 case (ABMCA nomenclature) as representative of the POrbM cases (Fig. 2a–e; Fig. S1a; Table S1). The POrbM projects on the *basal* radial domain, mainly on its *medial basolateral* subdomain (blm-i', blm-i"; Fig. 2a–e). Along a series of rostrocaudal coronal sections, the projections could be followed through all the different strata of this radial subdomain: we first see (rostrally) terminals at the inner intermediate stratum, blm-i' (Fig. 2b–d), which then extend onto the outer intermediate stratum, blm-i" (Fig. 2c, d), ending finally (caudally) both at the superficial stratum, blm-s (the latter corresponds to a region of the classic posterolateral cortical amygdalar area, PLCo; Fig. 2c, d), and the periventricular stratum, blm-p (Fig. 2e). The *lateral*, *anterior*, and *posterior* radial domains, together with the *lateral basolateral*, and *basomedial* radial subdomains, did not receive practically any projections from the POrbM in any of their different strata (lat-i; lat-p; bll-i; bll-s; bll-p: ant-i, ant-s; post; Fig. 2a–e). The subpallial posterodorsal subdivision of the medial amygdala did receive projections from POrbM (MePD; Fig. 2b–d; see Fernández et al. [6]), while, in contrast, the pallial *medioventral* amygdala did not show POrbM inputs (mev; Fig. 2b–d). The contralateral amygdala presents a similar pattern of inputs (mainly in blm), but the projection is much weaker (Fig. S1a).

Next, we analyzed as representative of *n* = 9 injections in PL (Table 1), all studied section-by-section, the case PL-0.391 (Fig. 2f–j; ABMCA nomenclature; Table S1). The projection pattern is similar to that described for POrbM, with predominant inputs on the different strata of the blm radial subdomain (Figs. 2g-j). In this case, we observed also projections on the *medioventral* amygdala pallial subdomain (mev, Fig. 2g, h). The contralateral amygdala showed a similar projection pattern (Fig. S1b).

The *n* = 11 Allen cingulate mesocortex injections studied section-by-section that project to the pallial amygdala were placed mostly in rostral cingulate regions (Fig. 2k–m; Table 1; Table S1). The pattern in our representative case, ACAv-0.334 (ABMCA nomenclature) resembles that observed out of the POrbM and PL cortices, with predominant projections onto the periventricular and intermediate strata of the blm subdomain (blm-i'; blm-p; Fig. 2l, m). No clear labelling was observed in the corresponding superficial stratum. We also observed rostral Cing inputs onto the contralateral blm domain (Fig. S1c).

**Fig. 3:**
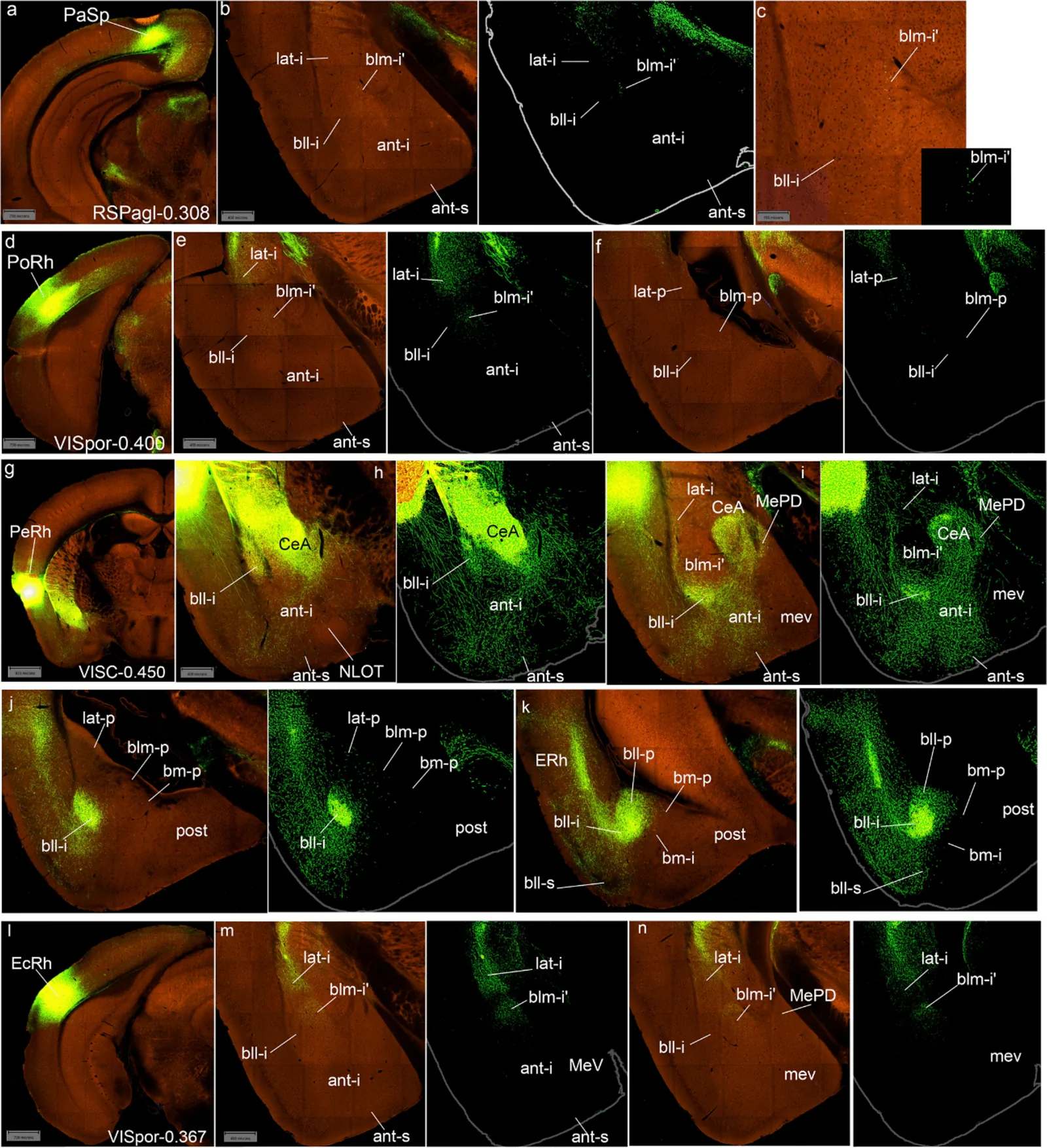
PaSp, PoRh, PeRh and EcRh projections to pallial amygdala. **a** PaSp injection site, experiment RSPagl-0.308. **b**–**c** PaSp projections over pallial amygdala in coronal sections from rostral to caudal. **d** PoRh injection site, experiment VISpor-0.400. **e**, **f** PoRh projections over pallial amygdala in coronal sections from rostral to caudal. **g** PeRh injection site, experiment VISC-0.450. **h**–**k** PeRh projections over pallial amygdala in coronal sections from rostral to caudal. **l** EcRh injection site, experiment VISpor-0.367. **m**, **n** EcRh projections over pallial amygdala in coronal sections from rostral to caudal. The material was obtained from the Allen Brain database (https://brain-map.org/our-research/connectivity), we used two-photon tomography and projection segmentation images. The scale bars were indicated in images

Projections from PaSp (*n* = 6 cases studied section-by-section) into the pallial amygdala were comparatively scarcer than the previously described ones (Table 1; Table S1). In our representative case (RSPagl-0.308; ABMCA nomenclature), the weak inputs reached mainly the blm subdomain (blm-i'; Figs. 3a-c). We did not detect PaSp inputs to the contralateral amygdala. There were no PoSp projections onto the pallial amygdala in the only Allen case that sampled this diminutive area at the back of the retrosplenial cortex (*n* = 1; not shown; Table S1). Injections performed at caudal levels of the mesocortical ring, affecting the PoRh cortex (*n* = 2, Table 1; Table S1), labelled the *lateral* radial domain as well as blm, possibly due to spread of the injection into visual cortex (e.g., case VISpor-0.400; ABMCA nomenclature; lat-i, lat-p, blm-i'; Fig. 3d–f). The contralateral amygdala showed similar but weaker labelling (not shown). The two cases available centered mainly on mesocortical PoRh may have affected visual associative isocortex apart of PoRh, as occurred also for the EcRh; this possibly explains the labelling upon the *lateral* domain in these cases.

The PeRh (*n* = 4 cases studied section-by-section, Table 1; Table S1), our representative case VISC-0.450 (ABMCA nomenclature; Fig. 3g–k; Fig. S1d) showed rostral projections that reached the intermediate stratum of the bll subdomain (bll-i; Fig. 3h, i). As we moved caudally along the coronal sections, inputs to this radial subdomain were maintained, reaching its superficial and periventricular strata (Fig. 3j–k). Similar projections were observed in the contralateral amygdala (Fig. S1d). Regarding the blm subdomain, it appears to receive some projections in its inner and outer intermediate strata, and periventricular stratum (blm-i', blm-i'', blm-p; Fig. 3i, j). The *anterior* radial domain also received abundant PeRh projections, both in its intermediate and surface strata (ant-i, ant-s; Fig. 3h, i). This contrasts with clear absence of PeRh projections on the mev pallial domain (Fig. 3i), which is molecularly very similar to the *anterior* complex [6]. The *lateral* radial domain also received projections in its most rostral intermediate stratum (lat-i; Fig. 3h, i). The bm radial subdomain and the *posterior* radial domain do not receive projections from the PeRh cortex (bm-p; bm-i; post; Fig. 3j, k). We did find abundant PeRh inputs on the subpallial amygdala, namely on the central nucleus and rostral regions of MePD (CeA: MePD; Fig. 3h–k).

In the cases analyzed section-by-section with injections in EcRh, the disgranular/granular subdivision of PeRh (*n* = 5, Table 1; Table S1), the projection pattern is completely different from that described for PeRh. This may be because the injections were very caudal, and/or may have invaded the visual isocortex (ICxV; ABMCA nomenclature; Fig. 3l). The representative case chosen was VISpor-0.367 (ABMCA nomenclature; Fig. 3l–n). In this case, we observed a projection to the intermediate stratum of the *lateral* radial domain, as well as to the inner intermediate stratum of the blm domain (lat-i, blm-i'; Fig. 3i, j). A similar but weaker projection was observed contralaterally (not shown).

**Fig. 4:**
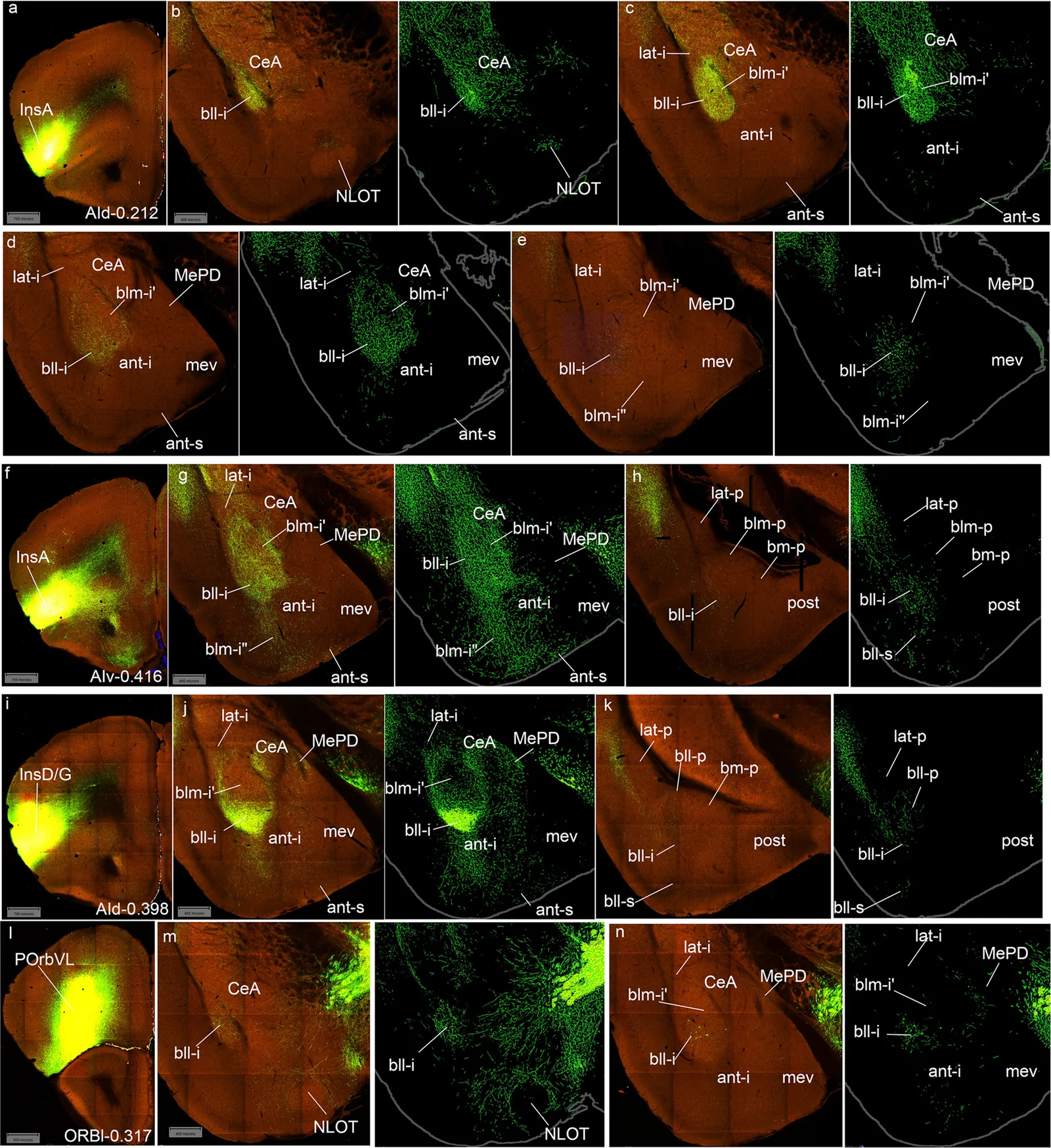
Agranular and disgranular/granular Ins and POrbVL projections to pallial amygdala. **a** InsA injection site, experiment AId-0.212. **b**–**e** InsA projections over pallial amygdala in coronal sections from rostral to caudal. **f** InsA injection site, experiment AIv-0.416. **g**, **h** InsA projections over pallial amygdala in coronal sections from rostral to caudal. **i** InsD/G injection site, experiment AId-0.398. **j**, **k** InsD/G projections over pallial amygdala in coronal sections from rostral to caudal. **l** POrbVL injection site, experiment ORBl-0.317. **m**, **n** POrbVL projections over pallial amygdala in coronal sections from rostral to caudal. The material was obtained from the Allen Brain database (https://brain-map.org/our-research/connectivity), we used two-photon tomography and projection segmentation images. The scale bars were indicated in images

For injections in the insula region (*n* = 5 cases studied section-by-section, Table 1; Table S1), we illustrate two cases (AId-0.212 and AIv-0.416) injected in the agranular region (Fig. 4a–h; Fig. S1e). In case AId-0.212, the insular inputs reach the intermediate stratum of bll, mainly rostrally (bll-i; Fig. 4b–e). The inner intermediate stratum of blm (blm-i') also received insular projections, mainly at rostral levels (Fig. 4b–e). Other radial domains and subdomains of the amygdala barely received insular projections in this case (Fig. 4b–e). However, in the case AIv-0.416 we observed predominant insular projections over the intermediate and superficial strata of the bll subdomain, in a distribution similar to what was described for PeRh (Fig. 4f–h). We also observed agranular insular inputs on the inner region of the blm subdomain, as well as in the *anterior* radial domain (blm-i', blm-i'', ant-i, ant-s; Fig. 4g). In both cases illustrated, we did not observe projections of AIv onto the *lateral* and *posterior* radial domains, nor onto the bm subdomain and mev (Fig. 4b–h). On the other hand, the subpallial amygdalar CeA distinctly received projections from InsA (Fig. 4b–d, g). To study the disgranular/granular insula, we used AId-0.398 as a representative case (Fig. 4i–k). The results obtained were very similar to those described for the agranular insula case AIv-0.416. All the Ins cases showed similar contralateral projections (Fig. S1e). In the PeRh and Ins cases, we did not analyze cases showing mainly claustral injections.

Finally, in rostrolateral regions of the mesocortical ring, we studied injections in the ventrolateral posterior orbital cortex (POrbvVL; *n* = 9 cases studied section-by-section, Table 1; Table S1) and chose ORBl-0.317 (ABMCA nomenclature) as a representative case (Fig. 4l–n; Fig. S1f). We observed POrbVL projections on the intermediate stratum of the bll subdomain (bll-i; Fig. 4m, n). The rest of the pallial amygdala domains and subdomains did not receive significant inputs from this mesocortical sector. Scarce comparable projections were detected in the contralateral side (Fig. S1f).

The Results thus showed on the whole that the mesocortical ring projects primarily onto the bll and blm parts of the *basolateral* subdomain of the *basal* radial domain. Medial regions of the ring, such as the POrbM, PL, and Cing cortices, project onto the blm, while lateral regions of the ring (PeRh, Ins, POrbVL) project onto the bll. The PeRh and insular cortices also project extensively onto the *anterior* radial domain, additionally to their bll projection.

### Isocortical Associative Projections onto the Pallial Amygdala

Next, we examined the projections of the isocortex onto the pallial amygdala. As previously published, it is the associative cortices (not the primary ones) that project to the amygdala (reviewed in Pitkänen [12] and Sha et al. [13]).

**Fig. 5:**
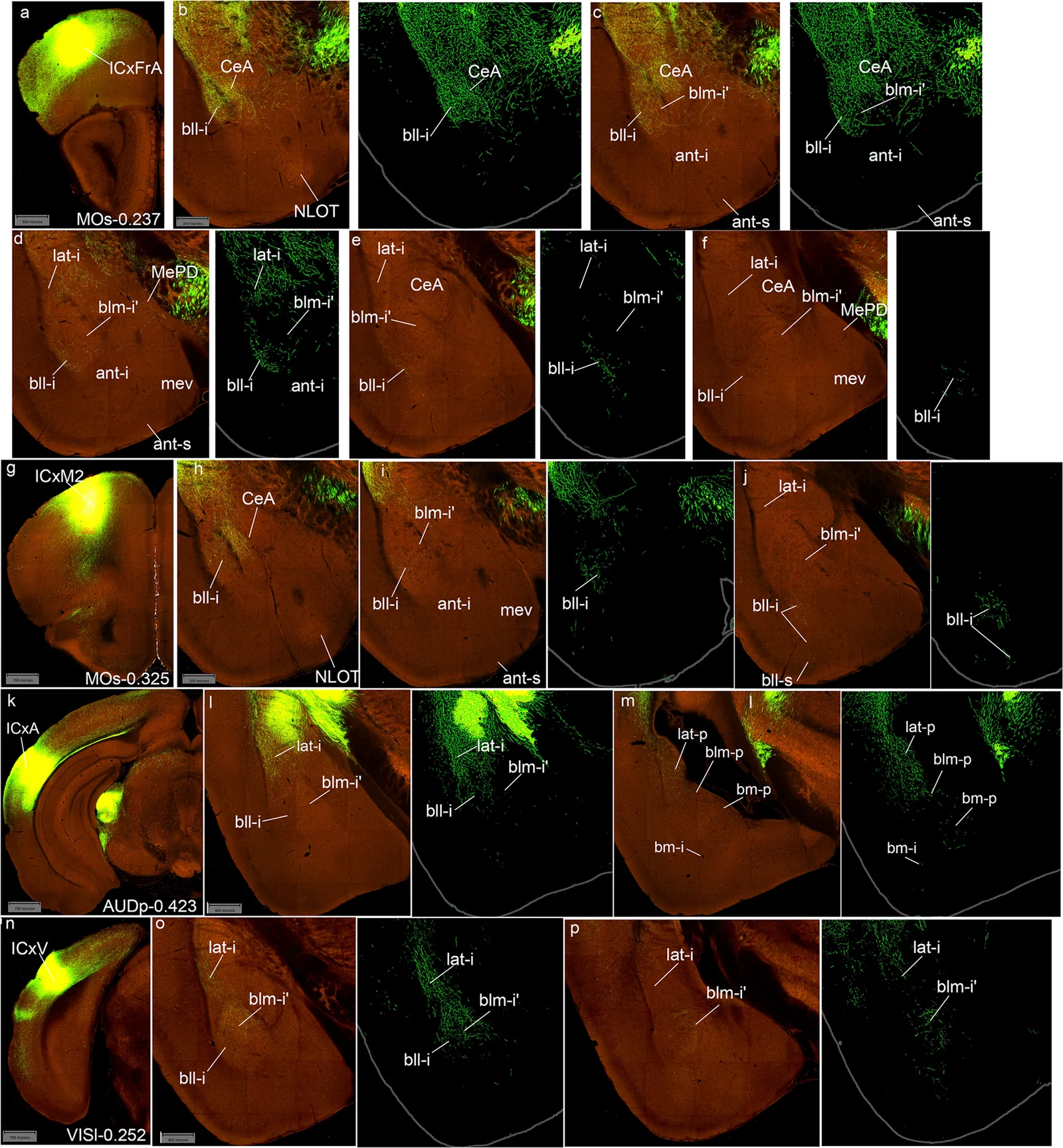
Prefrontal, motor, auditory and visual associative isocortical projections to pallial amygdala. **a** Prefrontal ICx injection site, experiment MOs-0.237. **b**–**f** Prefrontal ICx projections over pallial amygdala in coronal sections from rostral to caudal. **g** Motor ICx injection site, experiment MOs-0.325. **h**–**j** Motor ICx projections over pallial amygdala in coronal sections from rostral to caudal. **k** Auditory ICx injection site, experiment AUDp-0.423. **l**, **m** Auditory ICx projections over pallial amygdala in coronal sections from rostral to caudal. **n** Visual ICx injection site, experiment VISl-0.252. **o**, **p** Visual ICx projections over pallial amygdala in coronal sections from rostral to caudal. The material was obtained from the Allen Brain database (https://brain-map.org/our-research/connectivity), we used two-photon tomography and projection segmentation images. The scale bars were indicated in images

As a representative ABMCA case of those injected in the associative frontal cortex (ICxFrA; *n* = 3 cases studied section-by-section, Table 1; Table S1), we analyzed case MOs-0.237 (ABMCA nomenclature Fig. 5a–f; Fig. S1g). In this experiment, we observed amygdalar terminals starting at the rostralmost region of the intermediate bll subdomain (bll-i, classic BLA; Fig. 5b,c). The following sections show an extension of the label into deeper parts of the intermediate bll, including the laterally adjacent intermediate part of the lat domain, bordered by the amygdalar capsule, that also received some projections at rostral levels (lat-i, bll-i; Fig. 5d–f). Other regions of the pallial amygdala do not receive projections from the frontal association cortex. However, the subpallial central amygdalar nucleus does receive inputs from ICxFrA (Fig. 5b–f). A similar projection pattern was detected contralaterally (Fig. S1g).

Regarding the projections of the motor association cortex (ICxM2) onto the pallial amygdala, we studied MOs-0.325 as a representative case (Fig. 5g–j; *n* = 6 cases studied section-by-section, ABMCA nomenclature, Table 1; Table S1). We observed a labelling similar to the frontal cortex case, with terminals reaching the most rostral region of the intermediate bll, but also the lat intermediate stratum and more caudal areas and the corresponding superficial bll stratum (lat-i; bll-i; bll-s; Fig. 5j). The deep intermediate stratum of blm hardly receives any projections (Fig. 5i). Similar projections were detected also in the contralateral bll (not shown).

We next analyzed cases of projections from the auditory association cortex onto the pallial amygdala (Fig. 5k–m; Fig. S1h; *n* = 4 cases studied section-by-section, Table 1; Table S1). We illustrate AUDp-0.423 (ABMCA nomenclature) as a representative case. The projection from this experiment appears centered on the *lateral* radial amygdalar domain, specifically on its intermediate and periventricular strata (lat-i, lat-p; Fig. 5l,m). The radial bm subdomain also received some collateral projections along its radial dimension (bm; Fig. 5m). The bl subdomain receives few projections from this cortex, probably in cases where the injections spread to the adjacent mesocortical ring (bll-i; bll-p, blm-i'; Fig. 5l, m). Weaker labelling, although with a similar distribution pattern, was observed in the contralateral amygdala (Fig. S1h).

As a representative of the associative visual cortex, we illustrate the case VISl-0.252 (ABMCA nomenclature, Fig. 5n-p; *n* = 3 cases studied section-by-section; Table 1; Table S1). In this experiment, we observed an amygdalar projection similar to that described above for EcRh; the injection possibly was not strictly localized to ICxV. In any case, the *lateral* radial domain receives projections in its intermediate stratum, as does the deep intermediate stratum of the blm unit (lat-i, blm-i'; Fig. 5o, p). In this case, clear labelling was not detected in the contralateral amygdala. In other associative areas, the injection volume of all available Allen experiments was very low.

### Mesocortical Ring Projections Corroborate the Radial Structure of the Blm and Bll Amygdalar Subdomains

**Fig. 6:**
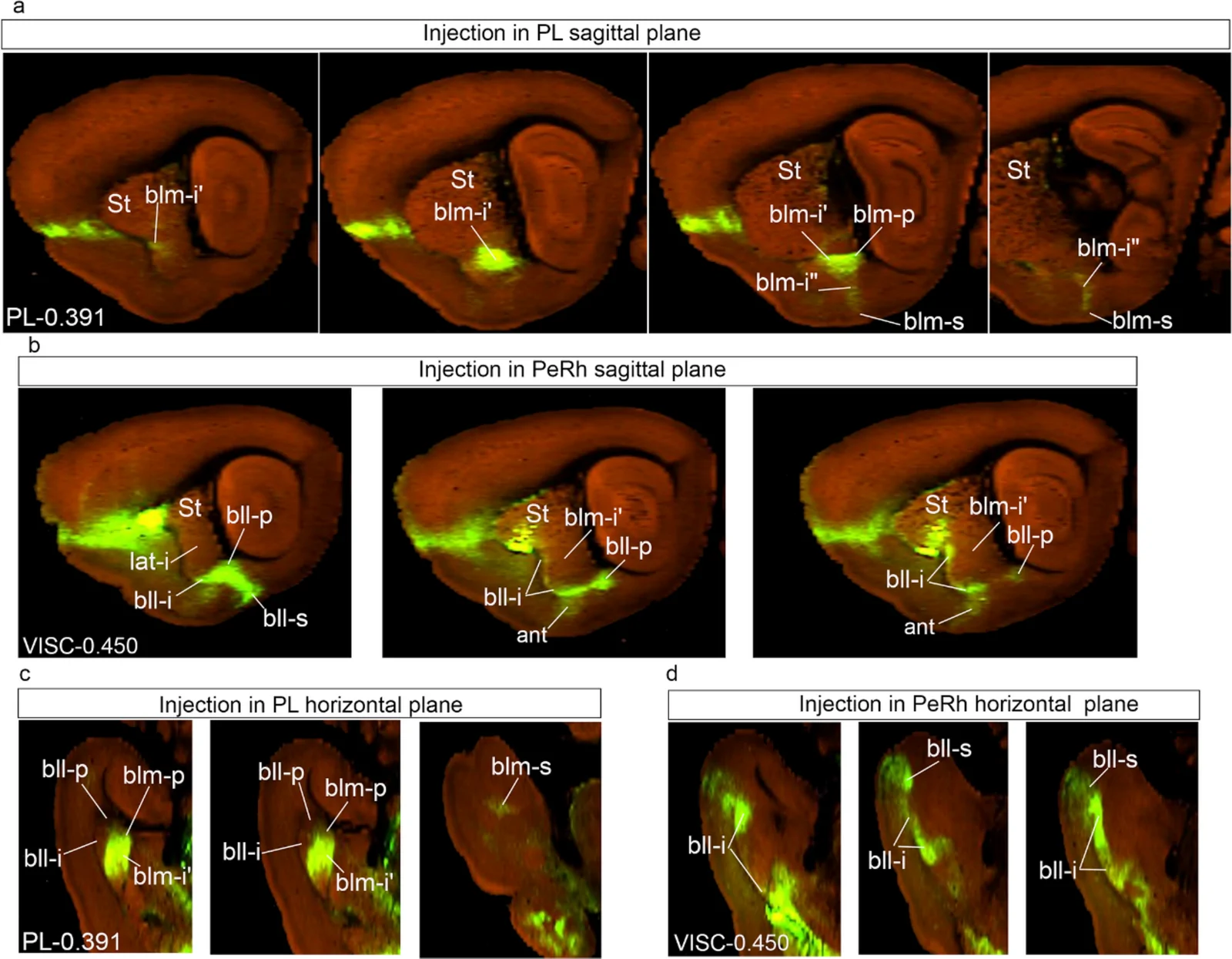
Projection patterns delineate radial domains and subdomains in the pallial amygdala. **a **Projection pattern of PL injection over pallial amygdala, its blm subdomain, experiment PL-0.391. Sagittal plane. **b** Projection pattern of PeRh injection over pallial amygdala, its bll and ant domain/subdomain, experiment VISC-0.450. Sagittal plane. **c** Projection pattern of PL injection over pallial amygdala, its blm subdomain, experiment PL-0.391. Horizontal plane. **d** Projection pattern of PeRh injection over pallial amygdala, its bll and ant domain/subdomain, experiment VISC-0.450. Horizontal plane

One of our aims was to test whether the terminal distribution of diverse cortical projections in the pallial amygdala was consistent with its postulated radial structure [4]. The projections of two studied cases, PL-0.391 (representing PL injections) and VISC-0.450 (representing PeRh injections) were reexamined in virtual sagittal and horizontal section planes obtainable at the AMBCA application (Fig. 6). This visualization mode enhances the visibility of the oblique amygdalar radial domains.

The PL cortex projections (case PL-0.391) reach the blm (Fig. 6a); they are observed across a small series of sagittal sections, delineating clearly the different nuclear strata of the blm radial subdomain: its intermediate (blm-i', blm-i''), periventricular (blm-p) and superficial (blm-s) strata. Similarly, the PeRh cortex projections upon bll (case VISC-0.450) delineate all the strata of the bll subdomain in the sagittal plane (bll-i, bll-p, bll-s; Fig. 6b). The virtual horizontal section plane provides a complementary view of the marked radial ventriculo-pial distribution of both cortical projections upon the blm (Fig. 6c) and bll domains (Fig. 6d; compare the genoarchitectonic identification of these radial domains reported in Garcia-Calero et al. [4], their Figs. 3, 5, 7).

**Fig. 7:**
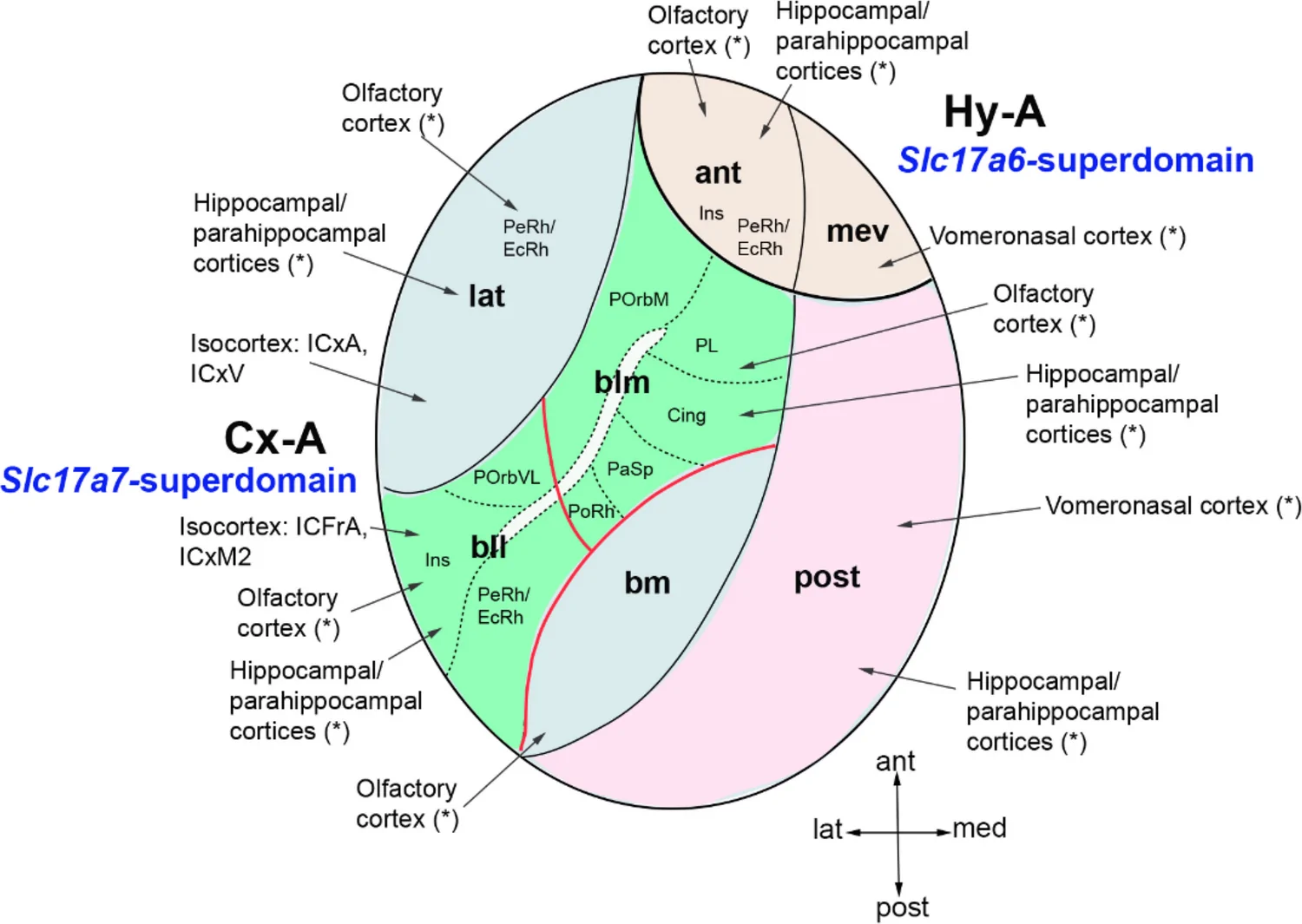
Cortical projections onto a topological map of pallial amygdala supported by transcriptomic data. Schema of the diverse cortical projections projected onto a topological flatmap of the pallial amygdala, subdivided in its main radial units: Hy-A and Cx-A, main molecular superdomains; lat, ant, mev and post, four radial histogenetic domains surrounding the basal domain; bll, blm and bm, the three subdomains of the basal radial domain. The mesocortical and isocortical projections were described in the present work. (*) Olfactory, vomeronasal, hippocampal and parahippocampal projections were extracted from the literature [10, 11]. Orientation: ant/post, anteroposterior axis; med/lat mediolateral axis

## Discussion

Recent transcriptomic studies of our lab [5, 6] indicate that the pallial amygdala is indeed divided into radial units and these are grouped into two molecularly distinct superdomains, identified as cortex-like (Cx-A) and hypothalamus-like (Hy-A). The difference lies in their differential expression of the two forms of the vesicular glutamate transporter *Slc17a7* (Cx-A) and *Slc17a6* (Hy-A)*.* The Hy-A portion shows the earliest neuronal birthdates [14], suggesting a differential evolutionary origin. The *basal*, *lateral,* and *posterior* radial units belong jointly to the Cx-A superdomain, whereas the *anterior* and *ventromedial* radial units belong to the Hy-A superdomain (Fig. 7). In this report, we mainly deal with the *basal* and *lateral* units, with occasional reference to the other units.

As regards the Hy-A superdomain, present data indicate that the *anterior* radial unit (comprising the classical BMA and ACo nuclei) receives an evaluated separate copy of mesocortical gustatory, interoceptive and auditory information from the Ins and PeRh regions. Previous studies link this region with evolutionarily old circuits processing olfactory, taste and interoceptive sensory inputs vehiculated via the parabrachial nucleus, or the olfactory and insular cortices, and producing outputs into the hypothalamus [15–18].

In contrast, as regards the Cx-A superdomain, it allows comparison of the topographical connections upon it of the entire mesocortical ring versus those of the isocortex. In the past, these two cortical amygdalar input sources were not sufficiently distinguished from each other (e.g., Hintiryan et al. [9] classified all mesocortical areas as ‘isocortex’) or were examined only partially (i.e., isolated mesocortical areas were studied -most frequently, the cingulate, insular or orbitofrontal ring sectors-). Often, the subliminal notion contemplated in the antecedent literature was that cortical input to the amygdala was essentially *sensorial/perceptive* in nature (i.e., informing about the circumstances of the experience, consistently with a purely isocortical origin), rather than resulting from previous *evaluation* of the experience; evaluation was supposed to emerge within the amygdala. We contrarily hold that limbic evaluation probably occurs essentially in the mesocortical ring, thanks to its intrinsic integrative connectivity [8] and its singular differential (synaptic) molecular profile. The latter suggests a computing algorithm distinct from that of the isocortex, at least within upper layer mesocortical neurons. We postulate that the amygdala essentially connects its perceptive background isocortical input with the evaluated mesocortical input and then apparently distributes the combined result to different cortical and subcortical targets.

The functional analysis sketched above emerges from our own molecular definition of the mesocortical type [1], jointly with our recent observation that the mouse mesocortex ring integrates topographically varied limbic processing via multiple mutual interconnections of the diverse ring sectors [8]. This suggested a previously unappreciated integrative role for this type of cortex, potentially useful in emotional evaluation, motivation and behavioral modulation. Moreover, the present Results indicate that the massive afferent projection from the mesocortical ring is the main limbic cortical input to the Cx-A amygdala superdomain, while the separate isocortical input obviously has a complementary perceptual role. The full mesocortical ring targets mainly the whole *basolateral* radial subdomain of the *basal* unit (see flatmap representation in Fig. 7). Furthermore, this connectivity exhibits an orderly topographic distribution: the medial regions of the ring, comprising the POrbM, PL, Cing, PaSp and PoRh cortices, project onto the *medial* subdivision of the *basolateral* unit (blm), whereas the lateral POrbVL, Ins, and PeRh sectors project mainly onto the *lateral* subdivision of the *basolateral* complex (bll), maintaining their relative order within the cortical ring. In addition, the mesocortical-amygdalar projection is molded along the predicted radial dimension of blm and bll (Fig. 6a, b), corroborating them as functionally unitary in relation to specific sectorial mesocortex projections, as a result of their neurogenetic derivation from molecularly distinct amygdalar progenitor domains [4–6, 14].

The parallel projections from the associative isocortex (i.e. visual, somatosensory and auditory cortex projections) target mainly the *lateral* radial complex. Their sensory nature probably indicates that they have less *limbic* weight and a different functional role compared to the mesocortical inputs. Olfactory allocortex also projects onto the pallial amygdala, reportedly onto the early-born superficial strata of all radial domains [19, 20] (Fig. 7). Parahippocampal/hippocampal inputs onto selected parts of the amygdala across deep, intermediate and superficial strata were also described [9–12, 19, 20] (Fig. 7); these inputs possibly mediate memory consolidation and/or spatial maps.

The importance of the *basolateral* radial amygdalar subdomain (bl) as the main receptor of limbic mesocortical ring projections is underlined by its main output pathways via its *lateral*, bll, and *medial*, blm, subdivisions (classically known as basal, B, basolateral, BL, or anterior basolateral, BLA, complex [9–13, 20–30].

1. Feedback to the mesocortical ring, namely its orbitary, prelimbic, anterior cingulate, insular and perirhinal sectors, and potentially also to the additional parasplenial and postrhinal sectors added recently by us [1]. Note that in the literature, these mesocortical targets are usually misinterpreted as isocortical areas (e.g., [9]).
2. Projection to subpallial centers, namely the central amygdalar nucleus (medial subdivision), and the intercalated nuclei of the amygdala. These project to hypothalamus, periaqueductal gray, and pontine nuclei and are considered direct and indirect pathways, respectively, for fear/anxiety responses and aversive behavior.
3. Projection to the striatal subpallium (ventral striatum and olfactory tubercle, including the nucleus accumbens), implicated in reward-related behavior, and its dorsal caudate-putamen subdivision, implicated in instrumental learning.
4. Projections to the hippocampal and parahippocampal cortices and the medio dorsal thalamic nucleus (the latter projects to the orbitary cortex).

Some recent publications indicate segregation of connectivity patterns between the two subdivisions of the *basolateral* unit. For instance, Beyeler et al. [24] performed retrograde tracer injections in nucleus accumbens and the central amygdalar nucleus. They observed an ordered medio-lateral projection from the classic basolateral nucleus (BLA; our bll and blm) to these two subpallial areas. Medial BLA (blm) cell populations project mainly to nucleus accumbens and relate preferentially to reward predictions. In contrast, cell populations located in the lateral BLA (bll) project to the central amygdalar nucleus, which apparently generate aversive predictions. Their schematic Fig. 3c [24] resembles our *blm* and *bll* radial subdivisions of the *basolateral* complex (Fig. 7) that receive preferentially projections from *medial* versus *lateral* parts of the mesocortical limbic ring (the lateral insular cortex is known to be related to aversive evaluations [31]).

Injections in PeRh, PoRh and EcRh were observed to produce also terminal labeling in the *lateral* radial unit, though it is unclear how far these injections may have overlapped associative visual or auditory cortex. The *lateral* unit also receives thalamic auditory and pain sensory inputs and is implicated in conditioned fear response through long-term potentiation [32–35]. The *lateral* domain projects on the *basolateral* amygdalar domain and also back to the PeRh and PoRh mesocortex [9–13, 20, 21, 26, 35, 36] (and Allen brain data). It projects also to the lateral subdivision of the central amygdalar nucleus, considered to initiate another pathway for fear response [22].

We thus postulate that high-level, associative but *non-evaluated* perceptual and motor planning correlates originated in the isocortex follow a dual pathway into the amygdala: a direct isocortical-amygdalar route into the *lateral* unit (whose output converges thereafter into the bl) and an indirect *evaluative* and probably slower isocortical-mesocortical-amygdalar route that massively reaches the *basolateral* amygdalar node. The POrbM that participates in this pathway represents a hierarchically most significant cortical limbic hub due to its bidirectional connections with the entire mesocortical ring [8, 37].

The amygdalar bl may act as a downstream output relay station for previously cortically integrated limbic evaluations. We think that the necessarily complex limbic evaluation may be strictly a task that needs multiple interconnected cortical structures rather than a nuclear function. Nevertheless, a potential collateral modulatory role of the amygdala in some aspects of evaluation itself may depend on the details of intrinsic radial connections within the diverse amygdalar units and their mutual horizontal interconnections. This analysis suggests that the isocortical input relayed into the bl by the *lateral* amygdalar domain possibly acts complementarily introducing additional algorithms into the parallel limbic computations. In addition, the expansion of the lateral nucleus of the pallial amygdala in primates is probably related to the expansion of the isocortex [38].

### Future Directions

Finally, we conjecture that the POrbM hub afferent to the amygdalar bl node probably integrates the partial complementary evaluations generated in the apparently antagonistic medial and lateral parts of the mesocortical ring (reward versus aversion; note many real situations may need both aspects covered simultaneously, as we know from introspective experience) and may also address significant results into the hippocampus for memorization. Reciprocal connections between posterior and medial orbitofrontal cortex and basal amygdala have been described in primates previously [39–41]. One important potential result of POrbM function over a lifetime might be the synthetic, persistent and slowly changing *Self* construct, a psychological function which is already widely contemplated in the literature but has so far remained devoid of an anatomical correlate candidate. The possibly *Self-*related POrbM area connects mainly with the *medial basolateral* (blm) bl subdomain, making this amygdalar region an important hub in the limbic connectome.

## Supplementary Information

Below is the link to the electronic supplementary material.

Supplementary file1 (XLSX 15.1 KB)

Supplementary file2 (DOCX 1.15 MB)

## Data Availability

To develop the present work, we used the Allen Brain Mouse Connectivity Atlas (https://brain-map.org/our-research/connectivity).

## Glossary

ant: *anterior *radial domain
ant-i: *anterior *radial domain, intermediate stratum
ant-s: *anterior *radial domain, superficial stratum
bll: *lateral basolateral* radial subdomain
bll-i: *lateral basolateral* radial subdomain, intermediate stratum
bll-p: *lateral basolateral* radial subdomain, periventricular stratum
bll-s: *lateral basolateral* radial subdomain, superficial stratum
blm: *medial basolateral* radial subdomain
blm-i': *medial basolateral* radial subdomain, deep intermediate stratum
blm-i": *medial basolateral* radial subdomain, superficial intermediate stratum
blm-p: *medial basolateral* radial subdomain, periventricular stratum
blm-s: *medial basolateral* radial subdomain, superficial stratum
bm: *basomedial *radial subdomain
bm-i: *basomedial *radial subdomain, intermediate stratum
bm-p: *basomedial *radial subdomain, periventricular stratum
CeA: central amygdala
Cing: cingulate cortex
ICxA: auditory associative isocortex
ICxFrA: prefrontal associative isocortex
ICxM2: motor associative isocortex
ICxV: visual associative isocortex
Ins: insular mesocortex
InsA: agranular insular mesocortex
InsD/G: disgranular/granular insular mesocortex
lat: *lateral *radial domain
lat-i: *lateral *radial domain, intermediate stratum
lat-p: *lateral *radial domain, periventricular stratum
MePD: posterodorsal medial amygdala
mev: *ventromedial* radial domain
PaSp: parasplenial mesocortex
PeRh: perirhinal mesocortex
PL: prelimbic mesocortex
POrbM: medial posterior orbitary mesocortex
POrbVL: ventrolateral posterior orbitary mesocortex
PoRh: postrhinal mesocortex
PoSp: postsplenial mesocortex
post: *posterior* radial domain
St: striatum
