# Supplementary material for: Topographic Mesocortex-Amygdalar Projections Expand the Molecular and Functional Model of the Limbic System
Source: Mol Neurobiol. 2026 Aug 25;63(1):856. doi: 10.1007/s12035-026-06153-8 (PMC13506595; doi:10.1007/s12035-026-06153-8)
Supplement: Supplementary file 1 [file 12035_2026_6153_MOESM2_ESM.docx]

**Supplementary Information**

**Molecular Neurobiology**

**Title: Topographic mesocortex-amygdalar projections expand the molecular and functional model of the limbic system**

Luis Puelles^1,2^, Gloria Fernández ^1,2^ and Elena Garcia-Calero^1,2^*

^1^Departamento de Anatomía Humana y Psicobiología, Facultad de Medicina, Universidad de Murcia, Campus de Ciencias de la Salud, 30120 Murcia, España.

^2^ Instituto Murciano de Investigación Biosanitaria IMIB-Pascual Parrilla, 30120 Murcia, España.

*Corresponding author:

e-mail:ecalero@um.es

<https://orcid.org/0000-0002-7184-9584>

**Fig. S1**

**Contralateral POrbM, PL, Cing, PeRh, Ins, POrbVL, motor and auditory projections to the amygdala**

Projections of the experiments injected into (a) POrbM (ORBm-0.350), (b) PL (PL-0.391), (c) Cing (ACAv-0.334), (d) PeRh (VISC-0.450), (e) agranular Ins (AId-0.212), (f) PorbVL (ORBl-0.317), (g) motor cortex (MOs-0.237) and (h) auditory cortex (AUDp-0.423), to the contralateral amygdalar side. The material was obtained from the Allen Brain database (<https://brain-map.org/our-research/connectivity>), we used two-photon tomography and projection segmentation images. The scale bar were indicated in image (a).

**Table S1**

Excel table containing information on the analyzed cases: case, cortical ring model nomenclature (Nomenclature c. ring model), experiment ID, mouse line, injection coordinates (AP,ML,DV,∠), injection volume (mm^3^), target volume (mm^3^), tracer type. Target structures: COA (cortical amygdalar area), PAA (piriform-amygdalar area), HATA (hippocampo-amygdalar area), LA (lateral amygdalar nucleus), BLA (basolateral amygdalar nucleus), BMA (basomedial amygdalar nucleus), PA (posterior amygdalar nucleus), TR (postpiriform transition area).

ISI: Intrinsic Signal Imaging.
